# Dopamine fast determination in pharmaceutical products using disposable printed electrodes modified with bimetal oxides carbon nanotubes nanocomposite

**DOI:** 10.1038/s41598-025-91675-9

**Published:** 2025-04-02

**Authors:** Hend S. Magar, El-shazly M. Duraia, Rabeay Y. A. Hassan

**Affiliations:** 1 Biosensors Research Lab, Zewail City of Science and Technology, 6Th October City, Giza, 12578 Egypt; 2 Applied Organic Chemistry Department, National Research Centre (NRC), Dokki, Giza, 12622 Egypt; 3 Physics Department, Faculty of Science, Suez Canal University, Ismailia, Egypt

**Keywords:** Dopamine analysis, Electrochemical detection, Nanostructured electrodes, Bimetallic oxides, Carbon nanotubes, Chemistry, Materials science, Nanoscience and technology

## Abstract

Dopamine is an essential neurotransmitter involved in the regulation of our pleasure, motivation, and other biological functions. Thus, tracking and monitoring the biological dopamine level is crucial for the rapid and effective treatment as well as for the diagnosis of the neurodegenerative and neurological disorders. Nanostructured electrochemical systems are tested and validated as promising methods for dopamine detection. In this study, carbon nanotube-anchored bimetallic manganese/copper bi-oxides nanocomposite-modified screen-printed carbon electrodes (Mn/Cu oxides @CNTs-SPCEs) were exploited for the electrocatalytic oxidation and direct determination of dopamine. From the morphological analysis, the particle size of the bimetallic oxides spherical nanoparticles was ranged from 9.0 to 45 nm, while the electrocatalytic activity of nanocomposite towards dopamine oxidation was examined by cyclic voltammetry (CV) and differential pulse voltammetry (DPV) to demonstrate the acquired high sensitivity and selectivity. The optimized DPV assay provided a wide linear dynamic range of dopamine concentrations (from 0.001 to 140 µM), and a low detection limit of 0.3 nM. Eventually, the newly modified electrochemical method was applied for dopamine detection in pharmaceutical products with high accuracy.

## Introduction

Dopamine, (4-(2-aminoethyl) benzene-1,2-diol), is playing an important role in our central nervous system. It is an essential neurotransmitter regulating our pleasure, motivation, and other biological functions^1^. Accordingly, controlling the dopamine level is very sensitive, hence irregular dopamine production may lead to various psychiatric disturbances, and different neurological disorders such as addiction, depression, and Parkinson’s disease. Thus, tracking and monitoring the biological dopamine level is crucial for the rapid and effective treatment as well as diagnosis of the neurodegenerative and neurological disorders^2–4^.

From the analytical chemistry point of view, several protocols were developed and applied for the detection of dopamine in various matrices including Liquid chromatography–mass spectrometry (LC-MS/MS)^5,6^, high-performance liquid chromatography (HPLC)^7^, resonance rayleigh scattering^8^, chemiluminescence (CL)^9^, and colorimetric and spectroscopic probes^10,11^. All those methods owned the sensitivity, selectivity and applicability. Nevertheless, several obstacles came up and must be solved: (a) dopamine analysis using the current mentioned methods takes a long time due to the long assay procedure. (b) Complicated sample pretreatments are needed. (c) Large size of the advanced equipment and they are not suitable for the direct, rapid, and onsite analysis. Hence, developing alternative methods to provide selective, sensitive, eco-friendly and direct convenient determination is needed.

Electroanalytical methods are being exploited in diverse applications, including biomedical analysis, quality control, industrial and environmental monitoring. The electroanalytical techniques provide high specificity, ultra-sensitivity with low limit of detections. Therefore, these techniques are widely used for redox reactions and biological interactions, and for the quantifications of bio-molecules concentration in different complex matrices^12–14^.

Thus, as a special classes of electroanalytical techniques, sensors^15–18^ and biosensors^19–24^ provided fast analysis and direct quantification for multiple small and large molecules in biological^25^, food^26,27^ and environmental samples^24,28,29^. Applying the electroanalytical methods and chemical sensors in fast analysis of biological and environmental analysis has a great impact on enhancing the routine analysis^30^. This enhancement is strongly supported by the electrode modification with nanomaterials, due to the acquired electrical conductivity, larger surface area and electrocatalytic activity ^31^. Hence, various nanostructured materials (magnetic nanoparticles^32^, carbon-based materials^33,34^, and metal^35^ or metal oxides^36^) were exploited for designing selective electrochemical assays for dopamine analysis^37^. These developed assays provided promising applications in disease monitoring, drug development^27^, and medical diagnosis^38–41^.

Previously, several enzyme-based biosensors were developed and applied for dopamine analysis providing high selectivity. However, low enzymatic stability, their complex immobilization processes restricted their progressions. On the other hand, antibody-based biosensors are rarely used for dopamine detection due to the difficulty for obtaining sensitive and fast detection needed for it in situ assays. Fortunate, direct electrochemical detection of dopamine using nanomaterial-based sensors without the use of selective biorecognition element(s), e.g. antibody or enzymes exhibited several important advantages. For instance, modified electrode surfaces with nanostructured material demonstrated tailored redox potential window to track a selective detection with high sensitivity and detection redox reaction out any prior sample pretreatments.

Despite the promising advances in developing nanostructured-electrodes for dopamine detection, there are still several challenges that need to be addressed to make these electrodes practical for clinical applications. Weak stability for the modified electrodes, especially in harsh conditions may lead to decrease in the assay accuracy and reliability. Furthermore, the high cost of producing nanomaterials make the electrodes expensive and limit their accessibility. Overcoming these limitations will support the establishment of sensing systems and facilitate their potential environmental and biological uses.

In this study, we avoided the use of complex machinery procedures to synthesize manganese-copper bimetallic oxide (Mn-Cu oxides @CNTs nanocomposite) to be exploited as a new surface modifier for dopamine electroanalysis. Incorporation of two-metal oxides with the carbon nanotubes offered high catalytic activity, and high surface area resulting in a rapid response time and stable performance. Accordingly, low detection limits, high sensitivity, and excellent selectivity for dopamine detection were achieved.

## Experimental and methods

### Materials and instrumentation

Potassium ferro cyanide K_4_[Fe(CN)_6_]·3H_2_O, potassium chloride (KCl) and potassium ferri cyanide (K_3_[Fe(CN)_6_], were obtained from Pio CHEM. Dopamine were purchased from Solarbio Life Science. Copper sulfate (CuSO4) and potassium permanganate (KMnO4), uric acid and ascorbic acid were obtained from Sigma-Aldrich (St. Louis, MO, USA). Hydrochloric acid (HCL) was obtained from Loba-Chemie PVT-LTD, India. All experiments and measurements were carried out in double distilled water (DDW). Phosphate buffer saline (PBS, tablets with the pH = 7.4) were purchased from MPBio. Screen-printed carbon electrodes with dimensions: 50 × 13 mm (h × w) were brought from Zensors Company, where the printed electrodes which consist of three parts including silver as reference electrode, carbon for working and counter electrodes (the diameter of working electrode is 3.0 mm of graphitic carbon). All other chemicals and solvents were of analytical grade and were used without further purification.

Field emission scanning electron microscope (SEM, Helios NanoLab 400, accelerating voltage of 10 kV, working distance of 4.1 mm, and a current of 0.34 nA) was implemented for the morphological features studies for the synthesized nanocomposite. Electrochemical measurements (cyclic voltammetry (CV), differential pulse voltammetry (DPV), and electrochemical impedance spectroscopy (EIS)) were carried out at room temperature using a portable potentiostat (PalmSens4, Netherlands).

### Chemical synthesis of Mn–Cu oxides @CNTs nanocomposite

The nanocomposite of Mn-Cu oxides @CNTs was synthesized according to previous report published by Fouda et al.^42^. Multiwall carbon nanotubes (MWCNTs) were synthesized through chemical vapor deposition (CVD) utilizing methane gas as the sole carbon source. Consequently, acidic suspension of the canon nanotubes (1.0 g of MWCNTs) was made in diluted hydrochloride (HCl). After 60 min at room temperature, the suspension was collected, and washed several times with deionized to neutralize the pH. The MWCNTs aqueous suspension was dried overnight in a vacuum oven at 60^O^C to modify the MWCNTs surface and to form functional groups on the CNTs surfaces. Consequently, equal masses of MWCNTs, copper sulphate (CuSO_4_) and potassium permanganate (KMnO_4_) were physically mixed in a mortar for 15 min to form a homogeneous solid mixture. This powder mixture was placed in a tube furnace and annealed in the open air for one hour at three different temperatures (from 250 to 450^ O^C by increasing step by 100^ O^C). The obtained black fine powder was collected, and characterized without any further modifications.

### Characterization of the newly synthesized materials

Field emission scanning electron microscope (SEM, Helios NanoLab 400, accelerating voltage of 10 kV, working distance of 4.1 mm, and a current of 0.34 nA) was implemented for studying the morphological features of the newly synthesized chemical composite. Further, electrochemical characterizations using the common techniques including the electrochemical impedance spectroscopy (EIS), and the cyclic voltammetry (CV) were applied for understanding identifying the acquired electrochemical properties. In this regard, a portable potentiostat (PalmSens4) connected with a three-electrode system of screen-printed carbon electrodes (SPCEs) was used for testing the electrochemical features. The surface area of each working SPCE was functionalized with the synthesized nanomaterials.

The EIS as well as CV experiments were carried out in three replications using the ferricyanide as the standard redox probe with the concentration of 5.0 mM. Potassium chloride (0.1 M KCl) was the corresponding supporting electrolyte. Each EIS experiment was conducted at a fixed frequency range (100000 to 0.1 Hz), at open circuit potential, under a 10 mV potential amplitude. While the CV measurements were recorded by cycling the potential from −0.4 to +0.7 V, with a scan rate of 50 mV/s. All EIS effective factors (Rs, Rct, Cdl, Warburg diffusion resistances, and capacitive phase elements (CPEs) were identified by fitting the collected Nyquist plots with equivalent Randless-circuits.

### Dopamine quantification using the modified working electrodes

An aqueous homogeneous dispersion of the characterized materials (5.0 mg/mL of the Mn-Cu oxides @CNTs nanocomposite) was prepared and ultra-sonicated for 60 min. Consequently, 10 μL was taken from the suspension and drop-casted onto the working area of the SPCE forming a thin layer of the deposited material. Differential pulse voltammetry (DPV) alongside the CV were applied for the determination of dopamine redox reaction as well as limit of the detection. CV measurements were recorded by cycling the potential from −0.4 to +0.7 V with a scan rate of 50 mV/s. DPV studies were performed with a potential range from 0.0 to −0.4 V. All the electrochemical tests were conducted at room temperature (25 ± 2 °C).

### Dopamine detection in pharmaceutical products

A freshly prepared Mn-Cu oxides @CNTs nanocomposite-SPCE sensor was applied for dopamine analysis in dose-form pharmaceutical products (Dopamine, Ibn Hayyan Pharmaceutical Industries and Dopamine, SUNNY MEDICAL) without any pretreatments. A certain volume (10 µL) of pharmaceutical products was added into the electrochemical cell containing 3.0 mL of the PBS. An optimized DPV assay (potential range of 0.0 to −0.4 V, scan rate of 50 mV/s) was applied for determination of the dopamine concentrations.

## Results and discussions

### Characterization of the nanocomposite

**Fig. 1:**
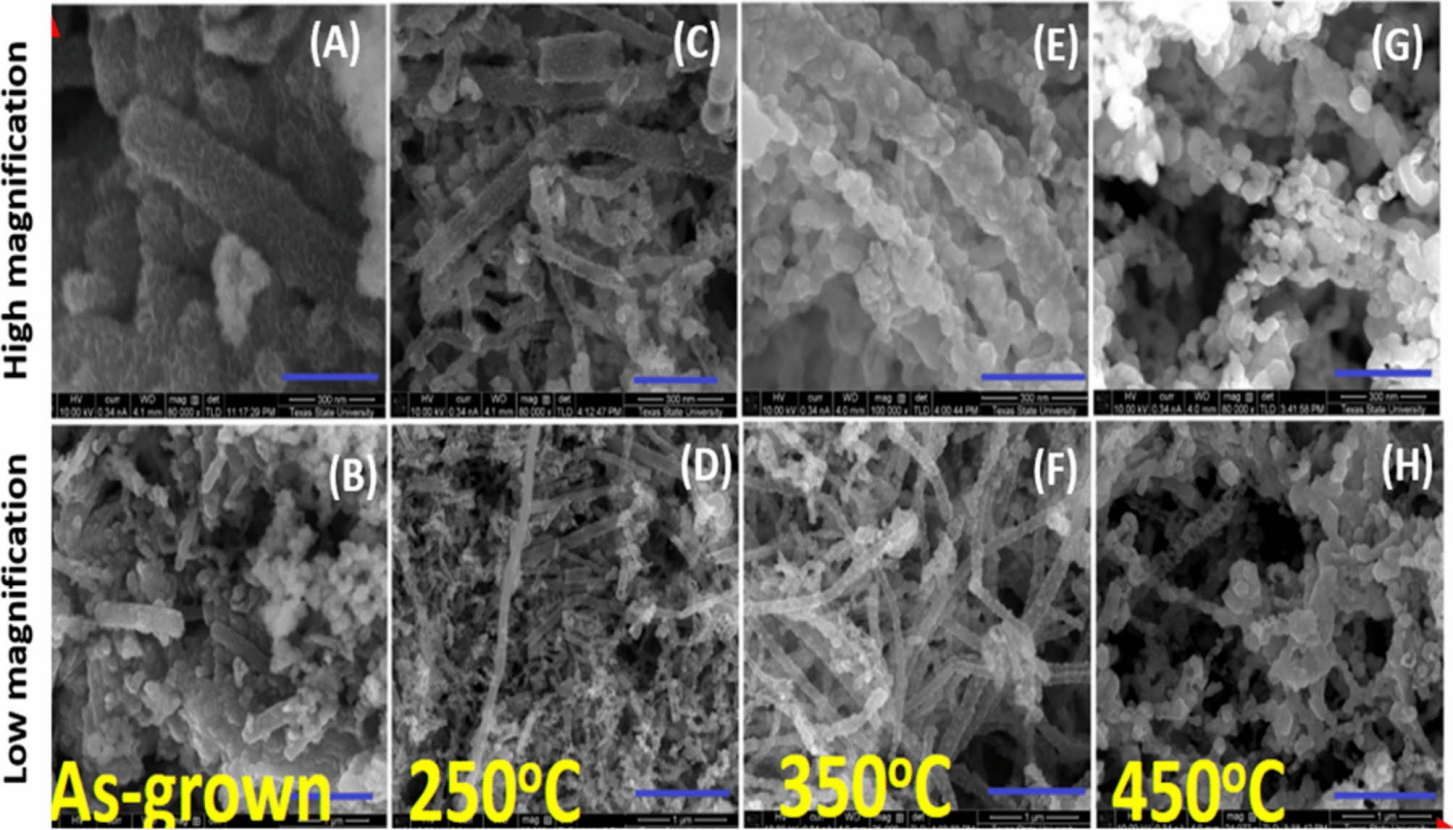
SEM images showing the attachment of Mn/Cu bi-metal oxides to the outer surface of carbon nanotubes. Two different imaging magnifications were used for the annealed samples (**A**,**B** as grown, **C**,**D** annealed at 250 °C, **E**,**F** annealed at 350 °C, and the **G**,**H** for the samples annealed at 450 °C).

Different nanomaterials were synthesized with different annealing temperatures (250, 350 and 450 °C). Accordingly, changes in morphologies and their relative electrochemical features were studied using the SEM, CV and EIS. As a result, the SEM analysis (see Fig. 1A–H) revealed the decoration of the outer surface of the CNTs with nanoparticles of the bi-metal oxides. The exhibited shape of the attached nano-spherical particles to the CNTs were irregular. The average particle size of the bi-metal oxides was ranged from 9.0 to 45 nm. However, a significant destruction effects were observed on the CNTs resulted from exceeding the annealing temperature to above 450 °C. This destruction effect is attributed to the thermal instability as well as the oxidation of the CNTs.

**Fig. 2:**
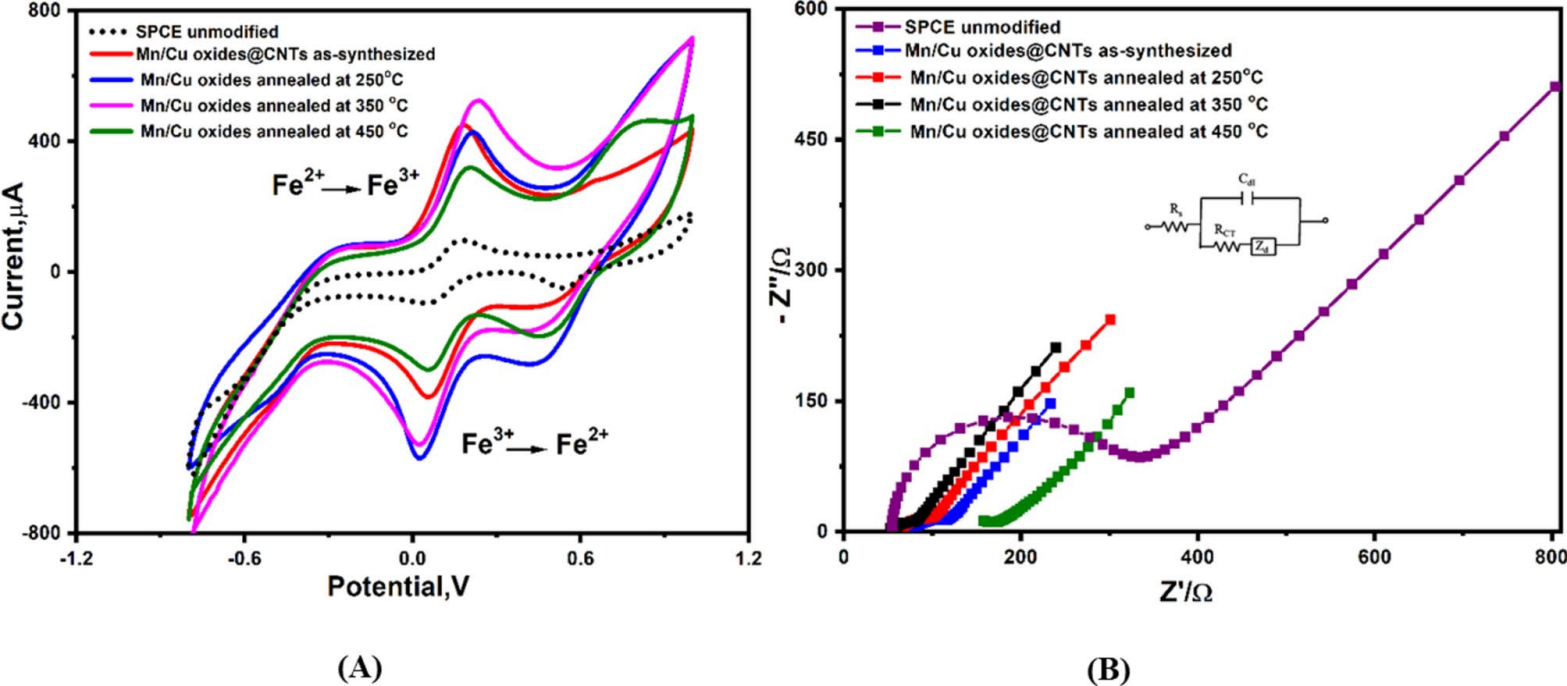
(**A**) Voltammetric characterization and (**B**) Nyquist plots of the impedimetric (EIS) characterization of modified SPCEs with the Mn/Cu oxides @CNTs composites annealed at different temperatures by using a solution containing ferricyanide (5.0 mM) and 0.1 M KCl. CV measurements carried out at scan rate of 50 mV/s (inset figure b represents the applied equivalent circuit for fitting the obtained Nyquist plots).

Subsequently, printed electrode surfaces of the SPCEs were modified with a thin film of each of the chemically synthesized nanomaterials before testing their electrochemical characteristics using the CV as well as the EIS. As a result, high Faradaic current was provided by the nanomaterial-based electrodes due to the fast and effective redox reactions of ferricyanide (the redox probe) at the electrochemically active surface area. Electrode modified with the annealed nanocomposite at 350 °C demonstrated the highest electrochemical performance, as shown in (Fig. 2A,B). Furthermore, electrochemical parameters extracted from (Fig. 2) are collected and presented in (Table 1). The increase in the anodic and cathodic peak currents (Ia, and Ic) were correlated with the increase in the annealing temperatures which is consistent with the particle size growth, resulted in an expansion in the electrochemically active surface area of the modified electrodes with the nanomaterials. From Nyquist plot, the observable decrease in the Warburg diffusion resistances due to the increase in the annealing temperature is attributed to the enhancement in conduction for larger grain of annealed samples.

**Table 1:** Extracted quantitative values of electrochemical parameters obtained from (Fig. 2A,B).

Electrode type	*I*_*pa*_* (µA)*	*I*_*pc*_* (µA)*	*E *_*oxd.*_* (V)*	*E*_*red.*_* (V)*	*R*_*s*_* (Ω)*	*R*_*ct*_* (Ω)*	*CPE n (µF)*	*W (Ω)*
Bare (unmodified)	103.2	−92.3	0.16	0.056	50.60	420.3	0.56 0.88	203.3
Mn/Cu oxides @CNTs	351.2	−303	0.32	-0.007	90.1	60.3	3.04 0.88	132.3
Mn/Cu oxides @CNTs annealed at 250 °C	484.2	−578.1	0.22	0.025	61.5	19.2	10.9 0.95	103.2
Mn/Cu oxides @CNTs annealed at 350 °C	536.2	−542.2	0.21	0.021	51.2	13.4	20.7 0.89	93.2
Mn/Cu oxides@ CNTs annealed at 450 °C	315.5	−294.2	0.199	0.46	56.5	109.2	1.21 0.89	163.2

*I*_*a*_, *I*_*c*_, *E*_*oxd*_, *E*_*red*_ are standing for the anodic peak current, cathodic peak current, potential of oxidation, and potential of reduction, respectively. While the *Rs*, *Rct*, *CPE* and *W* are standing for the solution resistance, charge transfer resistance, constant phase element, and Warburg resistance diffusion resistance, respectively.

### Scan rate effects on the redox reaction occurring at the modified electrodes

**Fig. 3:**
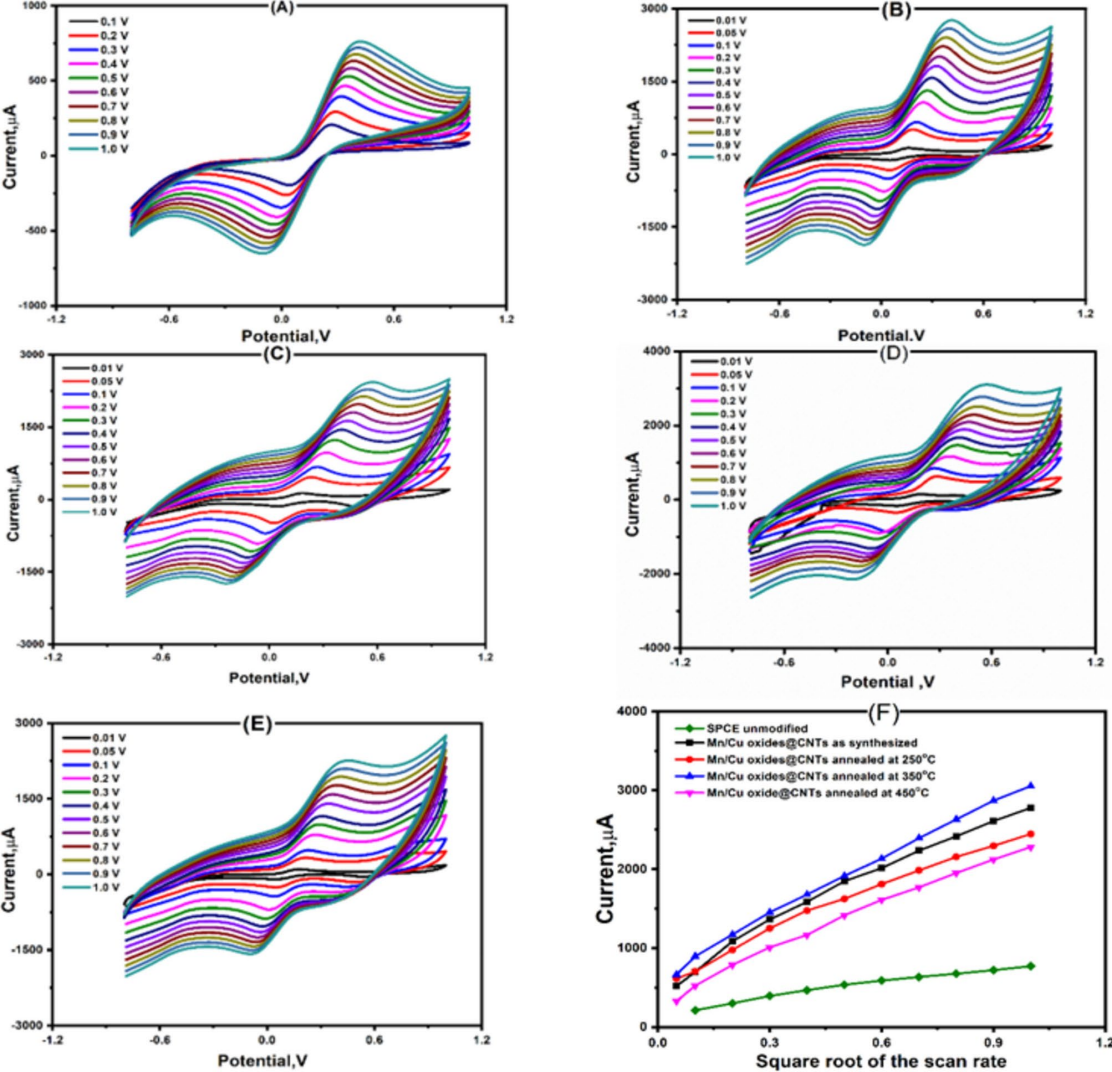
CV results showing the scan rate effect on the redox reactions of the FCN at the SPCEs (unmodified vs modified with the Mn/Cu oxides @ CNTs nanocomposites). Continues increase in the oxidation–reduction peak currents with the increase of the scan rate of (**A**) the bare electrode, (**B**) as-prepared nanomaterials, (**C**) annealed at 250 °C, (**D**) annealed at 350 °C, (**E**) annealed at 450 °C, and (**F**) all linear relationships with the square roots of scan rate.

To explore more about the electrochemical features of the modified SPCEs with the nanomaterials, voltammetric experiments were consequently performed at different scan rates in the ferricyanide (FCN) solution. As shown in (Fig. 3A–E), all used electrodes demonstrated a linear increase in the redox current while the applied scan rates were increasing. However, the highest generated linear redox reactions against the increase in the scan rate was produced by the Mn/Cu oxides @CNTs annealed at 350 °C, as shown in (Fig. 3E). Relying on the Randles–Sevcik equation^30^:

$$Ip = 2.69 \, * \, 10^{5} * \, n^{3/2} *A* \, D^{1/2} *Cu^{1/2}$$

where the *Ip, n, D, A, C,* and* u* are standing for the oxidation peak current (in amperes), the number of transferred electrons, diffusion coefficient (cm^2^/s), electrochemically active surface area (cm^2^), concentration of the FCN (Molarity), and the scan rate (V/s), respectively.

The anodic peak current (Ipa) and cathodic peak currents (Ipc) of the FCN redox reactions were plotted against the square root of the scan rate, as shown in (Fig. 3F). To that end, a good linearity with a correlation coefficient of 0.9966. Consequently, the effective electrochemically active surface area of unmodified vs the modified electrode surfaces with the Mn/Cu oxides @CNTs were calculated. Accordingly, a significant enlargement in the active surface area of all modified electrodes was achieved, whereas the obtained values of the unmodified SPCE was 0.251 cm^2^, while the surface areas of modified electrodes were 0.784, 0.865, 1.24 and 0.789 cm^2^ for the as-prepared, and the annealed materials at 250, 350 and 450 °C, respectively. Interestingly, the SPCE modified with the Mn/Cu oxides @CNTs annealed at 350 °C exhibited the highest expansion in the electrode active surface area (i.e. almost fivefold increase in order or magnitude).

### Dopamine redox reactions at modified electrode surfaces

**Fig. 4:**
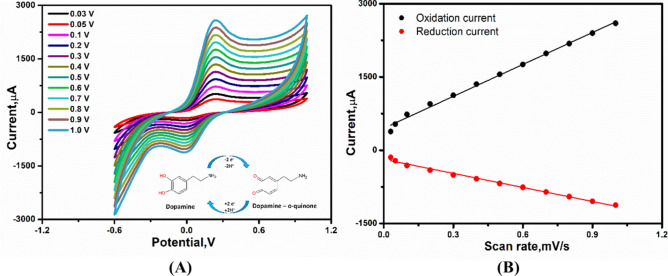
(**A**) Scan rate effects on the voltammetric redox reactions of dopamine at the modified printed electrodes (inset figure showing the redox reaction mechanism of dopamine). (**B**) Cyclic voltammograms showing the continuous increase of the redox peaks at different scan rates. Right side: Change in the oxidation/reduction peak heights at different scan rates.

The selected nanocomposite (Mn/Cu oxides@CNTs annealed at 350 °C) was used for the voltammetric analysis of dopamine at different scan rates ranging from 30 mV/s to 1000 mV/s (Fig. 4A). As a result, defined redox peaks were appeared in the range between −0.2 to 0.4 V showing the easiness of oxidation–reduction reactions of the targeting molecules at the designed nanostructured electrode surface, while a continued increase in the redox reaction of dopamine was dependent on the increase of the scan rate without the changes in the peak positions (see Fig. 4B), which confirm the diffusion-controlled process. The quasi voltammetric reactions of dopamine revealed the generation of two-electrons per each one-dopamine redo reaction, as shown in the inset of (Fig. 4A). The electrocatalytic reaction in dopamine involved two-protons and two-electrons transfer process during the redox reaction, as shown in (Fig. 4).

### Electro-catalytic oxidation of dopamine at the Mn/Cu oxides @CNTs nanocomposite

**Fig. 5:**
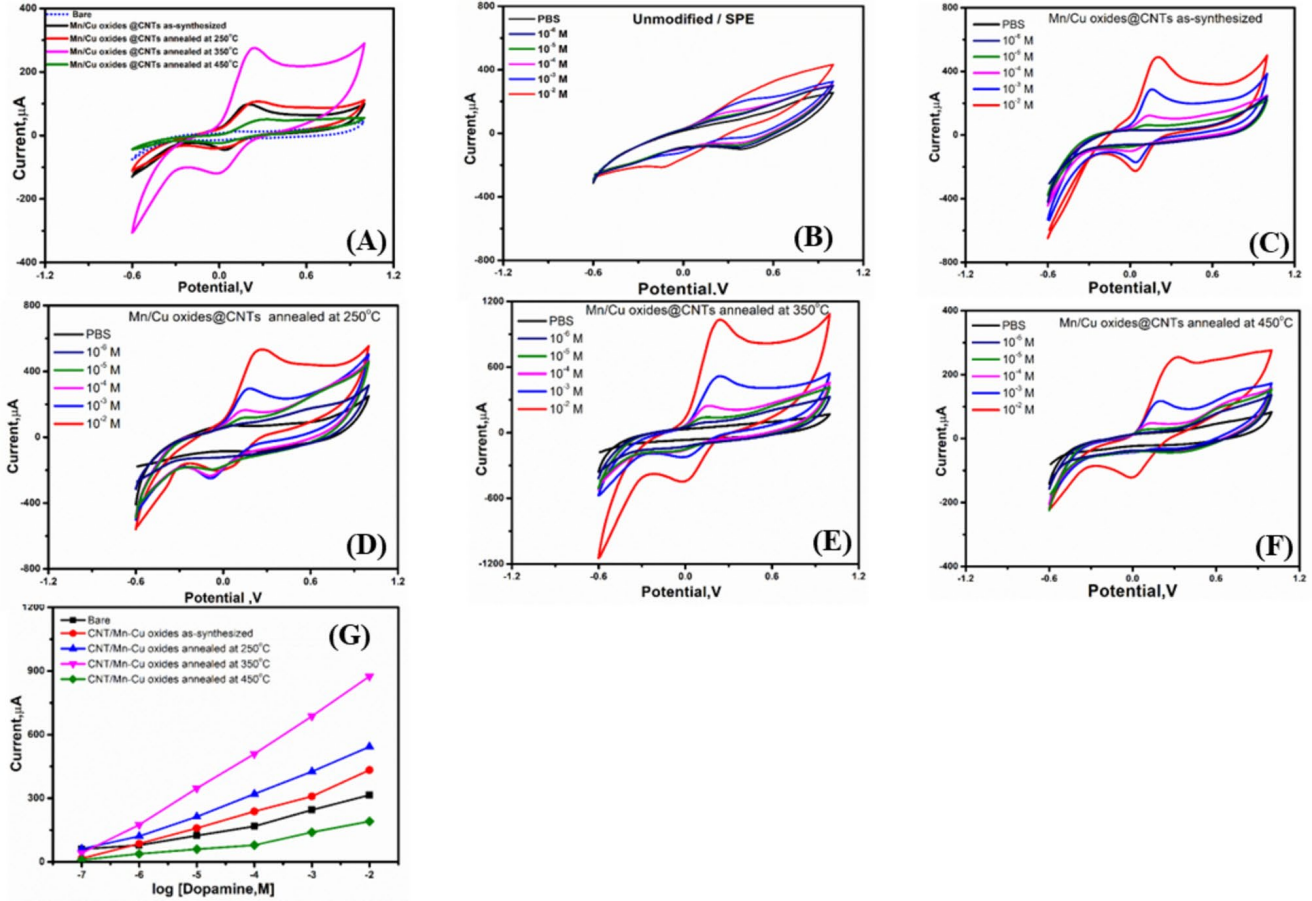
(**A**) Cyclic voltammetric responses of SPCEs (unmodified, or modified with the nanocomposite prepared at different temperatures) for 100 µM dopamine in PBS supporting electrolyte. Cyclic voltammetry curves for (**B**) unmodified, (**C**) Mn/Cu oxides @CNTs as-prepared materials, (**D**) Mn/Cu oxides @CNTs annealed at 250 °C, (**E**) CNTs annealed at 350 °C, (**F**) CNTs annealed at 450 °C. (**G**) Linearity curves of current vs. log dopamine concentration. The applied scan rate was 50 mV/s.

Following the nanomaterial characterization, the electro-catalytic behavior of the Mn/Cu oxides @CNTs nanocomposite was examined towards the electro-oxidation of dopamine. Dopamine has been successfully oxidized with a quasi-reversible peak at the unmodified surface of SPCE with a weak redox response, this weak voltammetric responses was totally improved after the electrode surface modification with the newly prepared nanocomposites (Fig. 5A). The oxidation peak current was found to be 67 µA for the bare SPCE, while the other modified electrodes exhibited much higher signals with 489, 543, 916 and 260 µA for the as-prepared materials, 250 °C annealing, 350 °C annealing, and 450 °C annealing, respectively. This means that the electrode modification with the nanocomposite enhanced the electrocatalytic oxidation of dopamine with more than tenfold of magnitude. This is a promising result to encourage building up an effective sensing system for the direct determination of dopamine. Based on the cyclic voltammetry measurements of synthesized materials as-prepared materials, 250 °C annealing, 350 °C annealing, and 450 °C annealing toward different concentrations of dopamine from 10^–6^ to 10^–2^ M (see Fig. 5B–F), the material annealed at 350 °C shows the highest sensitivity for detecting dopamine across different concentrations (as show in Fig. 5G). Consequently, the material annealed at 350 °C will be selected for further studies due to its enhanced performance at this annealing temperature.

### DPV determination of dopamine

From the sensitive quantitative analysis point of view, the CV is not the competitive voltammetric technique when it comes in a comparison with the differential pulse voltammetry (DPV). Thus, the CV cannot support the high sensitivity towards the determination of electroactive small molecules (e.g. dopamine). Therefore, the DPV is assigned and optimized in the next subsections.

From all the above discussed results, Mn/Cu oxides @CNTs annealed at 350 °C exhibited the remarkable electrochemical and electro-catalytic activities.

The DPV parameters are tested in order to find the best analytical signals of target analytes, the effect of pH of supporting electrolyte, accumulation time on the dopamine anodic peak current and selectivity were studied.

**Fig. 6:**
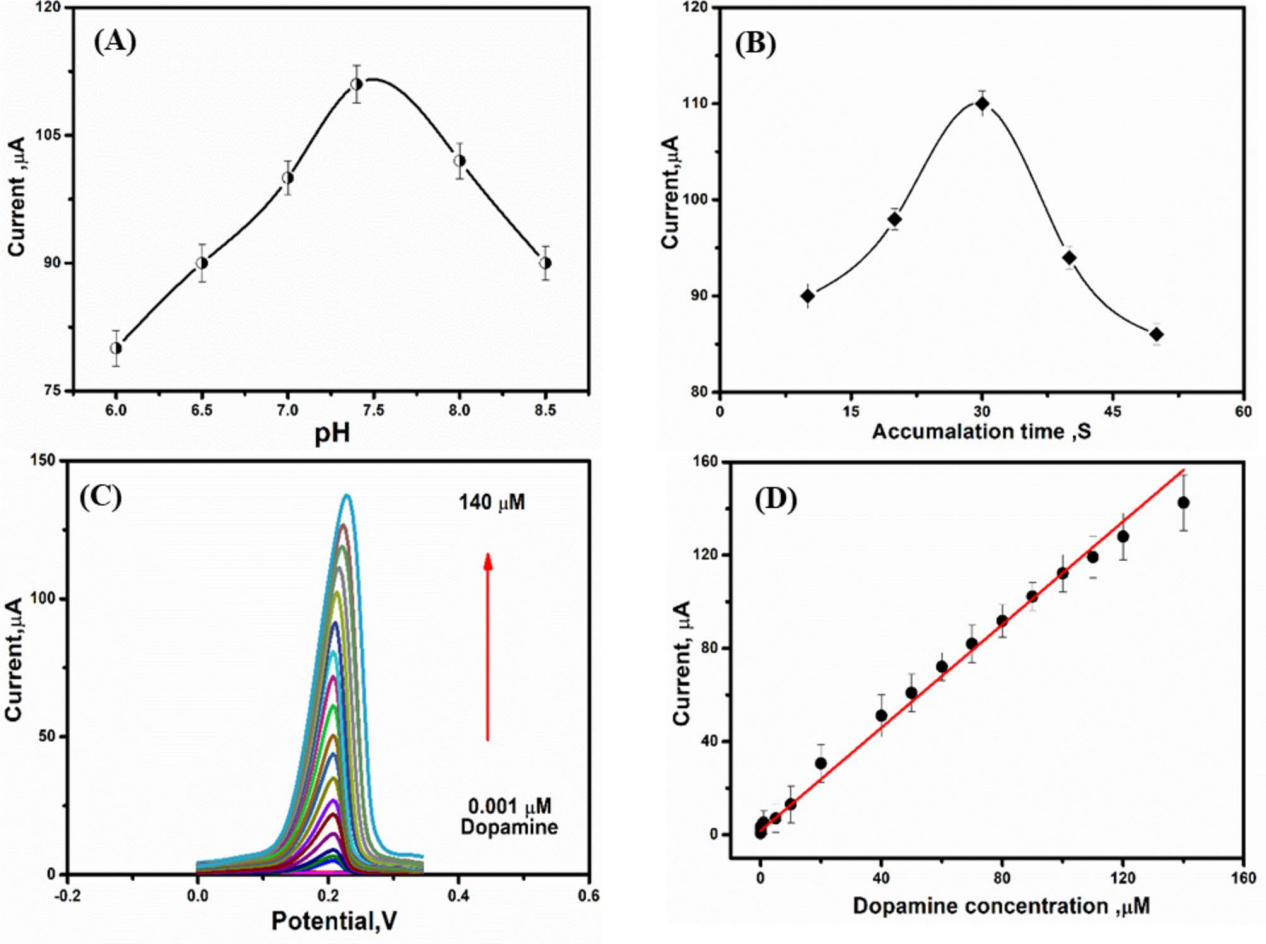
(**A**) bar diagram of pH values vs peak current. (**B**) linear plot of accumulation time vs. the current response. (**C**) DPV standard calibration curve of dopamine in PBS (pH 7.4), scan rate was 50 mV/s, and the deposition time was 30 s. (**D**) the DPV calibration plot of current vs. concentration (0.001 to 140 µM).

The supporting electrolyte pH has a significant effect on the electrochemical response of the proposed electrode toward dopamine detection. Therefore, PBS buffer with a different pH value between 6.0 -8.5 in the presence of 100 µM dopamine were tested for the electrocatalytic activity of Mn/Cu oxides @CNTs annealed at 350 °C, as shown in (Fig. 6A). The DPV peak oxidation current increased from 6 to 7.4 then decreased. Therefore, a PBS buffer at pH 7.4 was selected for all DPV electrochemical analysis of dopamine.

The peak current magnitude based on the electroactive species number which exist around the vicinity of electrode surface. Therefore, effect of accumulation time on the dopamine anodic peak current was studied by DPV technique at a scan rate of 50 mV/s by using Mn/Cu oxides @CNTs annealed at 350 °C modified SPCE in PBS buffer (pH 7.4) solution containing 100 µM dopamine. The value of oxidation peak current increased from 10 to 30 s then decreased (see Fig. 6B). The decrease in the peak current after 30 s may be owing to the saturation of dopamine at the electrode surface. Hence, 30 s is the best accumulation time which used for subsequent experimental work.

Thus, a calibration curve was obtained when at a wide dynamic range of dopamine concentrations (0.001–140 µM), as shown in (Fig. 6C). A strong linearity was obtained (see Fig. 6D) with a minimum standard deviation for each dopamine addition, and overall correlation coefficient R^2^ of 0.991. The assay limit of detection (LOD) and its limit of quantifications (0.3, and 0.6 nM) were calculated from the DPV calibration curve. Worth mentioning here that, this ultra-high sensitivity with low LOD was among the most sensitive electrochemical methods reported for the dopamine determination^43–50^, as depicted in (Table 2).

**Table 2:** Recently reported electrochemical methods for the direct determination of dopamine using modified working electrodes.

Electrode	Electrolyte	Method	Linear range, µM	Detection limit, µM	Ref.
Graphene/GCE	PBS (pH 7)	DPV	4–100	2.64	^43^
SWCNT/Fe_2_O_3_/GCE	PBS (pH 7)	SWV	3.2–31.8	0.36	^44^
Pt/RGO/MnO_2_/GCE	PBS (pH 7)	DPV	1.5–215	0.1	^45^
WO_3_/GCE	PBS (pH 7)	DPV	0.1–50, 50–600	0.024	^46^
NiHCF/PNH/AuE	PBS (pH 7)	DPV	0.1–4.3, 4.3–9.6	0.021	^47^
CuO nano-leaf	PBS (pH 8)	DPV	1–7.5, 7.5–140	0.5	^48^
ZnO nanorod/flower like CuO/AuE	PBS (pH 7.3)	CA	1000–8000	100	^49^
CuHCC nanocubes/GCE	PBS (pH 7.2)	DPV	0.1–350	0.019	^50^
Mn/Cu oxides@CNTs-SPCE	PBS (pH 7.4)	DPV	0.001–140	0.0003	*This work

*GCE* glassy carbon electrode, *AuE* gold electrode, *SPCE* screen-printed carbon electrode, *CA* chronoamperometry.

### The influence of interferences on the dopamine determination

**Fig. 7:**
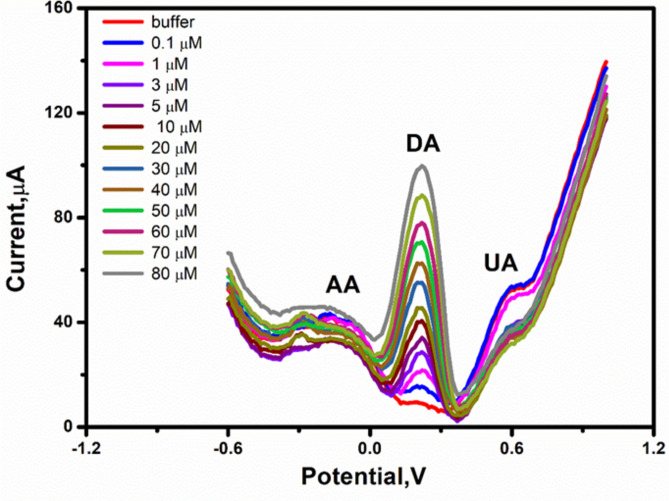
DPV determination of dopamine (DA) in the presence of ascorbic acid (AA), and uric acid (UA) using the SPCE modified with the newly prepared and selected Mn/Cu oxides @CNTs annealed at 350 °C. Each addition represents a mixture of the three compounds.

To test the selectivity features of the assigned DPV assay, several (foreigners) non-targeting molecules, other than the targeting compound (dopamine), were included in the DPV measurements. Ascorbic acid (AA) and uric acid (UA) are well known electrochemically active compounds that can easily be oxidized with voltammetric redox potentials close to the dopamine signals. In addition, the ascorbic and uric acids are present at higher concentrations when compared with the DA level in the human bodily fluids. Therefore, DPV responses of the dopamine were quantified with or without those two potential non-targeting molecules. As shown in (Fig. 7), the increase in the DPV signal was only dependent on the dopamine concentrations. However, no change in the DPV signal was observed from the non-targeting compounds. This result indicated the high selectivity features acquired by the newly developed DPV assay for the dopamine using the modified electrodes with the prepared nanocomposite.

### DPV analysis of dopamine in real samples

To study the practicability of the Mn/Cu oxides @CNTs nanocomposite-SPCE for the dopamine analysis in real samples, dopamine-HCl pharmaceutical injection products (200 mg/mL) were analyzed with standard addition method. An aliquot of each ampule was taken into the electrochemical cell, while the DPV was operated. Following the addition of dopamine solution from each sample, two standard additions took place. The total recovery of dopamine (97.0–101.0%) was calculated and tabulated as shown in (Table 3). This high accuracy is not limited to the dopamine analysis in pharmaceutical formulations. It is just a proof-of-concept for the simple determination of dopamine in real samples, and it opens the door for any other real sample analysis.

**Table 3:** DPV determination of dopamine in pharmaceutical formulations using the SPCE modified with the newly prepared nanocomposite.

Sample	Added (µM)	Detected (µM) ± SD	Recovery (%)
1	1	1.1 ± 0.1	110
2	10	9.8 ± 0.2	98
3	50	49.9 ± 0.1	99.8
4	70	69.9 ± 0.2	98.4
5	90	90.1 ± 0.1	100.1
6	110	109.8 ± 0.2	99.8
7	150	148.6 ± 0.3	99.06

## Conclusions

In this study, a novel nanocomposite made of Mn/Cu bi-oxides@CNTs was chemically synthesized, and characterized to be applied for the direct electrochemical detection of dopamine in pharmaceutical products. Screen-printed carbon electrodes (SPCEs) were used for the electrochemical characterizations (using the CV, and EIS), as well as for the DPV assay optimization and applications. Electrode modification with the newly synthesized nanocomposite exhibited high electrochemical performance, electro-catalytic activity and enabled the direct oxidation of dopamine. Over a wide dynamic range (from 0.01 to 100 µM), the DPV showed a linearity and ultra-high sensitivity with a limit of detection of 0.3 nM of the dopamine even in the presence of other electrochemically active molecules such as the ascorbic and uric acids confirming the selectivity of the optimized assay. Eventually, the dopamine was quantified in real samples, whereas high recovery percentages was achieved to prove the capability of using this sensing approach for tracking the dopamine level in real samples.

## Data Availability

Data are available upon request from the corresponding author of this article.
